# Supplementary material for: Clinical Phenotype and Management of Severe Neurotoxicity Observed in Patients with Neuroblastoma Treated with Dinutuximab Beta in Clinical Trials
Source: Cancers (Basel). 2022 Apr 10;14(8):1919. doi: 10.3390/cancers14081919 (PMC9026788; doi:10.3390/cancers14081919)
Supplement: Supplementary file 1 [file cancers-14-01919-s001.zip › cancers-1634922-supplementary.pdf]

Supplemental information on cases with severe CNS neurotoxicity

Case description and therapeutic interventions: For 27 patients (patients 1−10 LTI study, patients 11−19 HR-NBL1 R2 and patients 20−27 R4) detailed descriptions of clinical phenotypes, diagnostic findings, therapeutic interventions and outcomes are available and summarised in this report (**Table 3**). In one patient with tetraparesis, initially classified as peripheral neuropathy, the description of the clinical course of the neurotoxicity is not available and therefore not included below (Patient 19).

Patient 1 presented with dysphagia and inadequate reactions during Cycle 1 of dinutuximab beta (DB) with subcutaneous interleukin-2 (scIL-2) and isotretinoin (Cycle 1 [C1]/Day 13 [D13]). Immunotherapy was stopped, and one day later respiratory depression occurred, with periods of prolonged apnoea, paraplegia and fixed pupils in both eyes. The patient required endotracheal intubation and ventilation. Oedema of mesencephalon, pons, medulla oblongata and spinal cord was found on magnetic resonance imaging (MRI, Figure 2A). Mannitol and fluid restriction was used to treat the oedema and immunosuppressive therapy with dexamethasone (4 x 0.5 mg/kg/day) and intravenous immunoglobulins (IVIG) (1 g/kg/day for 2 days) was initiated. MRI performed 1 week later demonstrated an increase of the oedema, with lesions extending to crus posterius, thalami and capsula interna as well as genu and truncus corporis callosi. The patient's condition improved minimally, with only partially resolved symptoms. At the last follow-up (3 years after the event), the patient still presented with neuropathic bladder, and significantly disturbed motor activity and hand-eye coordination. The patient required ventilation support at home as well as nutrition via percutaneous entero-gastrostomy because he was unable to swallow.

Patient 2 developed fever and hypotension during Cycle 1 of DB and scIL-2 (C1D/8−12) requiring temporary discontinuation of DB. On Day 14 of the DB infusion (C1/D14), the patient complained of back pain. Several hours later (C1/D15), the patient experienced complete sensory and motor loss below T4/5, with loss of bladder function. MRI revealed myelitis of the thoracic and lumbar area of the spinal cord (Figure 1C). Immunotherapy was stopped. The patient had normal muscle

strength in upper extremities and normal cranial nerve function. The child was alert, orientated and had normal vigilance. In spite of immunosuppressive therapy (IVIG and high-dose dexamethasone, 30 mg/kg/day for 3 days), there was only minimal recovery, with no improvement 5 years after the event. Varicella zoster virus (VZV) infection on a thoracic dermatome was diagnosed 6 weeks prior to starting immunotherapy but had fully resolved before the start of the first DB infusion.

Patient 3 presented with VZV infection on the skin of the skull after the first round of scIL-2 during Days 1−5 of Cycle 1. Therefore, the combination treatment planned to start on C1/D8 was postponed and the patient received acyclovir therapy. After complete resolution of the lesions, the patient started to receive DB and scIL-2 two weeks later. On Day 7 of the DB infusion, the patient developed flaccid paraplegia and immunotherapy was stopped. Muscle strength of upper extremities was normal, with a decrease in muscle tone. Transverse myelitis was found on MRI (Figure 1B). Examination of cerebrospinal fluid (CSF) did not reveal any viral infection and polymerase chain reaction (PCR) for VZV was negative. Antibiotic (cefuroxime) and antiviral (acyclovir) treatment was given together with immunosuppression (steroids – first dexamethasone 3 x 0.5 mg/kg/day, then prednisolone for symptom progression of spinal cord injury: 30 mg/kg intravenous (i.v.) bolus, followed by 5.4 mg/kg/24 hours and IVIG 1 g/kg for 2 days and 0.5 g/kg for 2 days), but neurological symptoms deteriorated (hyposthenia of upper extremities, dysfunction of diaphragm). As symptoms progressed, plasmapheresis was initiated. The patient had 4 cycles of plasmapheresis during 7 days (Prismaflex, filter TPE 1000). The procedures were done using heparin anticoagulation, with 5% albumin and crystalloid fluid supplementation. The plasma exchange was 80 ml/kg/procedure. Rapid improvement of neurological symptoms was observed after the first plasmapheresis, with further regression of symptoms after the following procedures. The level of DB in the serum decreased from 5.8 µg/ml found at the time of event to 0.34 µg/ml after the last procedure. All neurological symptoms resolved and the patient is alive in 2nd complete remission 3 years after treatment.

Patient 4 presented with ataxia during the third cycle of DB combined with scIL-2 (C3/D17) and transverse myelitis was identified on MRI (Figure 1D). Immunotherapy was stopped. Symptoms progressed for two more days after cessation of treatment

with a complete loss of power in upper and lower extremities including pain and paresthesia. Spinal puncture revealed a slightly increased protein level with a normal leukocyte count. The DB level was 14.81 µg/ml in the serum, but no detectable levels were found in the CSF (0.02 µg/ml). The symptoms fully resolved after immunosuppression with IVIG and steroids. About 3 weeks before the event, the patient was diagnosed with a herpes simplex virus (HSV) infection and received a one-week course of acyclovir therapy until full resolution.

Patient 5 experienced urinary retention and hyposthenia during the first cycle of DB with scIL-2 (C1/D15). DB was stopped, and reintroduced at 50% of the dose two days after initial improvement of symptoms. However, symptoms recurred 4 hours after restarting DB. Immunotherapy was then discontinued and symptoms completely resolved after IVIG therapy (0.4 g/kg for 5 days). Examination of CSF was negative for DB. Sensorimotor demyelinating polyneuropathy with secondary axonal features, without conduction bloc was described in nerve conduction examinations. MRI revealed demyelinating neuropathy of dorsal roots.

Patient 6 initially presented with transient blurred vision in Cycle 1 of DB with scIL-2 (C1/D9), which resolved without intervention. The patient then developed capillary leak syndrome (C1/D13) and DB infusion was stopped. Two days later (C1/D15), urinary retention was observed, followed by paraplegia. On MRI, transverse myelitis was diagnosed and steroid therapy with dexamethasone followed by prednisolone was initiated. Examination of CSF was negative for DB. After treatment, symptoms of paraplegia disappeared, but 4 years after the event, the patient still suffers from neurogenic bladder and requires self-catheterisation.

Patient 7 experienced confusion and somnolence during the first cycle of DB with scIL-2 (C1/D9). Hypoxia (oxygen saturation 89%) was observed during the episode. An electroencephalogram (EEG) showed slowing due to encephalopathy. The symptoms completely resolved after DB cessation, without any additional treatment.

Patient 8 presented with urinary retention in Cycle 2 of DB with scIL-2 (C2/D11). scIL-2 and DB were discontinued, and symptoms completely resolved without

treatment. As morphine was stopped 3 days before the event, it was excluded as the possible reason.

Patients 9 and 10 presented with Grade 3 seizures and encephalopathy. No other data concerning the course, treatment or outcome are available.

Patient 11 presented with coma and paresis and was diagnosed as disseminated post-infectious encephalomyelitis. The event occurred during the treatment with scIL-2 in Cycle 4 and DB was not given because of prolonged fever after scIL-2 given on Days 1−5. Therefore, the last DB dose had been given 4 weeks before the event. As the drug level was expected to be >1 µg/ml, which is a level known to engage antibody-dependent cellular cytotoxicity (ADCC) and complement-dependent cytotoxicity (CDC) effector function of the antibody, a contribution of DB to this event cannot be ruled out. No infectious cause was found. The patient was treated with steroids, antibiotics and acyclovir, and symptoms resolved with sequelae.

Patient 12 experienced strabism for 2 days during Cycle 2 of DB (C2/D7) 2 days after completion of DB associated with palsy of the left 6th cranial nerve. Imaging studies revealed hyperintensive multiple lesions in the brainstem with suspected posterior reversible encephalopathy (PRES). Clinical symptoms resolved without sequelae after 1 month of oral prednisone treatment (25 mg/day) and did not reappear after reintroduction of DB at a reduced rate of 50%. Control MRI performed weeks later showed complete regression of previously described lesions.

Patients 13−16 were all treated with short-term infusion of DB combined with scIL-2 and isotretinoin and experienced seizures as the only neurotoxicity. One patient had fever, another one had a congenital central hypoventilation syndrome; there are no data concerning clinical status or laboratory examinations in the other patients. Two of them received a treatment rechallenge with DB only, with no symptoms recurring.

Patient 17 experienced mood disturbances followed by deterioration of the child’s general condition, severe motor weakness with refusal to walk, cachexia, dilated pupils with sluggish light reflex and photophobia. The impairment of taste started with refusal to eat. The patient was hospitalised for over 2 weeks and required total

parenteral nutrition. The event occurred during the first cycle of DB with scIL-2 and isotretinoin (C1/D9). Symptoms disappeared without treatment but the child needs eye glasses.

Patient 18 was diagnosed with PRES 3 days after the end of the last dose of DB and scIL-2 (scIL-2 was administered at 50% for persistent fever and hypotension).

Patient 19 was initially classified as peripheral neuropathy and the description of the clinical course of the neurotoxicity is not available.

Patient 20 presented with drowsiness on Day 10 of the first cycle of DB with scIL-2 and isotretinoin. In spite of morphine reduction, the neurological status worsened, with hypotonia and bulging fontanelle. Imaging studies revealed multiple focal areas of altered MRI signals of the white matter in both cerebral and cerebellar hemispheres. An infectious cause was excluded, and symptoms completely resolved after dexamethasone and IVIG.

Patient 21 was treated with DB and isotretinoin, and symptoms occurred after the end of the DB infusion in Cycle 3 (D11 blepharoptosis, D13 behavioral disturbances, self-inflicting trauma, blindness, torticollis, gait disturbances and hyperkinesis). MRI demonstrated encephalitis. The patient was treated with IVIG, methylprednisolone (2 mg/kg/day for 5 days) and acyclovir for 10 days with only transient improvement. One week after the occurrence of symptoms, the patient received 3 daily pulses of methylprednisolone (26 mg/kg/day) and 4 cycles of plasmapheresis performed on Days 21, 24, 26 and 28, with symptoms resolving. Neither HSV or VZV were found in blood and CSF.

Patients 22 and 23 were treated with long-term infusion of DB plus isotretinoin and experienced facial nerve paralysis. One of them had infiltration of the acoustic-facial package on MRI and was successfully treated with steroids, which resulted in the resolution of symptoms. The second patient had inflammation of mastoid cells on MRI and received antibiotics only.

Patient 24 experienced agitation with life-threatening behaviour; behavioural disturbances were observed since treatment beginning and reccurred after drug rechallenge. The symptoms resolved without sequelae.

Patient 25 experienced sensory disturbances in all extremities on Day 11 of Cycle 1 of DB with isotretinoin. Gait disturbances as well as problems with fine catch tasks in hands were observed, and sensory axonal neuropathy was diagnosed on MRI, with high protein levels in the CSF. The patient was treated with IVIG and steroids.

Patients 26 and 27 had severe neurological symptoms during DB plus scIL-2 and isotretinoin therapy. Patient 26 had seizures on Day 15 of Cycle 1, with no changes on MRI. The symptoms fully resolved and did not reappear in consecutive cycles. Patient 27 had gait disturbances (dragging left foot and walking with slightly broad-based gait) on Day 27 of Cycle 1. The symptoms fully resolved.

In summary, 12 patients received immunomodulatory treatment combinations of IVIG and/or steroids and in two cases, who did not respond to IVIG plus steroid, plasmapheresis resulted in complete resolution of symptoms. In eight patients, the CSF was examined without any specific findings. In two of the patients who had DB levels analysed in the CSF, DB was not detectable despite elevated levels found in the serum. In one patient, no detailed data are available.
